# Quantifying the relationship and contribution of mitochondrial respiration to systemic exercise limitation in heart failure

**DOI:** 10.1002/ehf2.13272

**Published:** 2021-02-20

**Authors:** Pim Knuiman, Sam Straw, John Gierula, Aaron Koshy, Lee D. Roberts, Klaus K. Witte, Carrie Ferguson, Thomas Scott Bowen

**Affiliations:** ^1^ Leeds School of Biomedical Sciences, Faculty of Biological Sciences University of Leeds Leeds LS2 9JT UK; ^2^ Leeds Institute of Cardiovascular and Metabolic Medicine University of Leeds Leeds UK

**Keywords:** Exercise, HFrEF, Lactate threshold, Skeletal muscle, V̇O_2peak_

## Abstract

**Aims:**

Heart failure with reduced ejection fraction (HFrEF) induces skeletal muscle mitochondrial abnormalities that contribute to exercise limitation; however, specific mitochondrial therapeutic targets remain poorly established. This study quantified the relationship and contribution of distinct mitochondrial respiratory states to prognostic whole‐body measures of exercise limitation in HFrEF.

**Methods and results:**

Male patients with HFrEF (*n* = 22) were prospectively enrolled and underwent ramp‐incremental cycle ergometry cardiopulmonary exercise testing to determine exercise variables including peak pulmonary oxygen uptake (V̇O_2peak_), lactate threshold (V̇O_2LT_), the ventilatory equivalent for carbon dioxide (V̇_E_/V̇CO_2LT_), peak circulatory power (CircP_peak_), and peak oxygen pulse. *Pectoralis major* was biopsied for assessment of *in situ* mitochondrial respiration. All mitochondrial states including complexes I, II, and IV and electron transport system (ETS) capacity correlated with V̇O_2peak_ (*r* = 0.40–0.64; *P* < 0.05), V̇O_2LT_ (*r =* 0.52–0.72; *P* < 0.05), and CircP_peak_ (*r =* 0.42–0.60; *P* < 0.05). Multiple regression analysis revealed that combining age, haemoglobin, and left ventricular ejection fraction with ETS capacity could explain 52% of the variability in V̇O_2peak_ and 80% of the variability in V̇O_2LT_, respectively, with ETS capacity (*P* = 0.04) and complex I (*P* = 0.01) the only significant contributors in the model.

**Conclusions:**

Mitochondrial respiratory states from skeletal muscle biopsies of patients with HFrEF were independently correlated to established non‐invasive prognostic cycle ergometry cardiopulmonary exercise testing indices including V̇O_2peak_, V̇O_2LT_, and CircP_peak_. When combined with baseline patient characteristics, over 50% of the variability in V̇O_2peak_ could be explained by the mitochondrial ETS capacity. These data provide optimized mitochondrial targets that may attenuate exercise limitations in HFrEF.

## Introduction

Heart failure with reduced ejection fraction (HFrEF) is a complex syndrome characterized by exercise intolerance and cardiac dysfunction. The severity of symptoms and exercise intolerance in HFrEF are most commonly assessed by the use of both subjective patient history (e.g. NYHA classification) and more objective laboratory‐based cardiopulmonary exercise testing (CPET). CPET is considered the gold‐standard assessment of exercise intolerance in patients with HFrEF,^1^, ^2^, ^3^ providing an integrated multi‐organ assessment. This facilitates refined non‐invasive assessment of key indices of exercise limitation that independently predict prognosis, which include peak oxygen uptake (V̇O_2peak_), oxygen uptake at the lactate threshold (V̇O_2LT_), and the ventilatory equivalent for carbon dioxide at the lactate threshold (V̇_E_/V̇CO_2LT_).^4^, ^5^, ^6^, ^7^, ^8^, ^9^, ^10^, ^11^

Although a defining feature of the HFrEF phenotype, the degree of left ventricular (LV) systolic dysfunction assessed by LV ejection fraction (LVEF) poorly correlates with the degree of exercise intolerance.^12^, ^13^, ^14^ In addition, pharmacological and device interventions that improve both LVEF and prognosis in HFrEF do not consistently improve exercise tolerance.^15^, ^16^ As such, a multi‐organ therapeutic approach in HFrEF has been advocated, which has identified peripheral skeletal muscle abnormalities as a key mechanism of exercise intolerance in HFrEF.^17^, ^18^, ^19^, ^20^, ^21^ Collectively, therefore, the need to clarify the relationship between skeletal muscle abnormalities and prognostic CPET measures in HFrEF could be useful for developing tailored therapeutic strategies aimed at improving exercise tolerance and quality of life.^22^

Of particular interest is the major therapeutic target of skeletal muscle mitochondrial dysfunction,^23^ which is proposed as a fundamental mechanism underlying persistent symptoms in HFrEF even when corresponding improvements in cardiac function are observed.^24^ Recent technological advances provide a window for mitochondrial function to be dynamically assessed across the electron transport chain using muscle biopsies from patients with HFrEF, which allows important novelty to be gained compared with past studies where static morphological/biochemical measures were only able to confirm global reductions in mitochondrial content correlated to impairments in V̇O_2peak_.^25^, ^26^, ^27^, ^28^ In contrast, the *in situ* interrogation of permeabilized myofibres allows evaluation of distinct respiratory states to identify key sites of limitation (i.e. across the electron transport chain at multiple mitochondrial complexes).^29^, ^30^, ^31^ In support, recent studies have confirmed that mitochondrial respiration from both the upper and lower limbs is reduced in patients with HFrEF, with impairments to complex I being closely correlated with V̇O_2peak_.^30^, ^31^, ^32^ Thus far, however, a comprehensive analysis of the relationship between mitochondrial respiratory states and key prognostic whole‐body CPET variables remains poorly explored in HFrEF, and there is limited information about the relative contribution of specific mitochondrial respiratory deficits to the degree of exercise limitation.

The present study, therefore, directly assessed skeletal muscle mitochondrial respiration from biopsies of patients with HFrEF in order to evaluate the relationship and quantify the contribution of distinct respiratory complex states to whole‐body prognostic CPET variables. We reasoned that a better understanding of the association between distinct mitochondrial deficits and whole‐body exercise variables would optimize future targets for attenuating symptoms related to exercise intolerance in HFrEF.

## Methods

### Participants

We approached consecutive patients with stable signs and symptoms of chronic heart failure (>3 months receiving medical therapy) that were untrained but ambulatory, and presented with a LVEF <50% measured by two‐dimensional echocardiography by Simpson's biplane following current guidelines^33^ who were listed for a cardiac implantable electronic device at Leeds Teaching Hospitals NHS Trust, as described in full details elsewhere.^30^ Participants were indicated for device therapy with either a pacemaker, implantable cardioverter defibrillator, or cardiac resynchronization therapy device according to current indications.^33^ Exclusion criteria for participation in the present study included the inability to provide informed consent due to cognitive dysfunction or the presence of comorbidities potentially confounding assessment of exercise intolerance such as other cardiovascular conditions, chronic obstructive pulmonary disease, or ongoing malignancy or limiting musculoskeletal disease. All patients provided written informed consent, and all procedures were conducted in accordance with the Declaration of Helsinki after receiving local institute ethical approval (11/YH/0291). The patients included in this study are a subset of those on whom we have previously published other outcomes.^30^, ^31^, ^32^

### Cardiopulmonary exercise test

Cardiopulmonary exercise testing data for patients were obtained from a peak symptom‐limited CPET conducted as part of routine clinical care, continued to volitional intolerance on a cycle ergometer for determination of V̇O_2peak_, V̇O_2LT_, peak circulatory power (CircP_peak_), and peak oxygen (O_2_) pulse, RER_peak_, and V̇E/V̇CO_2LT_. After a 5‐min warm‐up at 10 W, the patients started cycling at 20 W and workload was progressively increased by 5 W min^−1^ until the patients reached volitional intolerance. Ventilatory and pulmonary gas exchange variables were collected as 15 s averages on a breath‐by‐breath basis using a calibrated system (Ultimo CardO_2_, Medical Graphics, St. Paul, MN, USA). V̇O_2peak_ and RER_peak_ were recorded as the highest 15 s average, while V̇O_2LT_ was calculated using the V‐slope method, which also allowed V̇E/V̇CO_2LT_ to be determined.^34^ In the instance when a patient did not achieve lactate threshold and/or we were unable to estimate lactate threshold (*n* = 10), the V̇O_2LT_ and V̇E/V̇CO_2LT_ were not included in the analysis. CircP_peak_ was defined as the product of V̇O_2peak_ and peak systolic blood pressure^35^, ^36^, ^37^, ^38^ whereas peak O_2_ pulse was calculated as V̇O_2peak_ divided by heart rate peak. Patients removed the mouthpiece at the end of the test during recovery due to discomfort; VO_2_ and VCO_2_ recovery half time are therefore not reported.

### Muscle biopsy

Skeletal muscle biopsy of the *pectoralis major* (~50 mg) was obtained during the routine device implantation procedures as previously described,^30^ which took place within one month of baseline clinical data collection. There were no complications or adverse events with this procedure. A portion of muscle tissue was immediately placed in 1 mL of ice‐cold specialized preservation solution (BIOPS) for subsequent assessment of mitochondrial respiration.^31^ Recent evidence from patients with HFrEF indicate sampling of the upper limb muscles provide an opportunity to investigate the disease's systemic myopathy, with the *pectoralis major* providing a close surrogate for the vastus lateralis in terms of mitochondrial function and closely linked to whole‐body exercise intolerance that is not as impacted by other confounding factors (e.g. disuse, detraining, or arthritis).^30^ Furthermore, muscle mass of the *pectoralis major* is also a powerful predictor of prognosis in HFrEF^39^ and in relation to daily activities, the upper limbs typically perform various tasks (e.g. house chores and gardening) that would pose exercise limitations impacting patient quality of life. As such, the clinical relevance for sampling the pectoralis major in terms of exercise limitations and prognosis is well supported.

### Mitochondrial function

Mitochondrial respiration was measured *in situ* from saponin‐permeabilized skeletal muscle fibres using high‐resolution respirometry (Oxygraph‐2K; Oroboros Instruments, Innsbruck, Austria) as previously described elsewhere.^30^, ^31^, ^32^ Briefly, the following steps were performed including (i) Complex I leak respiration was determined by addition of glutamate (10 mM), malate (0.5 mM), and pyruvate (5 mM) (i.e. a measure of proton leak under non‐phosphorylating conditions); (ii) adenosine diphosphate (2.5 mM) was added to stimulate oxidative phosphorylation through Complex I (OXPHOS P_I_); (iii) outer mitochondrial membrane integrity was determined by addition of 10 μM cytochrome c; (iv) succinate at 10 mM to stimulate Complex I + II (OXPHOS P_I + II_); (v) 0.5 μM titrations of FCCP to achieve maximal uncoupled respiration for electron transport system (ETS) capacity; (vi) Complex I inhibitor rotenone at 0.25 μM for ETS‐supported Complex II respiration (C_II_); (vii) 2.5 μM antimycin A as a Complex III inhibitor for residual oxygen consumption (ROX) to calculate non‐mitochondrial (background) respiration, which was subtracted from all values. Complex IV respiration (C_IV_) was also measured by the addition of 0.5 mM TMPD and 2 mM ascorbate.^40^ Values for mitochondrial respiration were normalized to muscle wet mass and are presented as pmol O_2_/s/mg.

### Statistical analysis

Bivariate associations between mitochondrial and CPET variables were examined by Pearson's correlations coefficients. Multiple regression analysis was carried out to determine the independent contributors of V̇O_2peak_ variability. In a first step, variables with an established physiological role in V̇O_2peak_ including age, haemoglobin concentration (Hb), and LVEF were entered as a block to a multivariable regression model to determine how much of the variability in V̇O_2peak_ could be explained by these variables.^41^, ^42^, ^43^ We then ran the same model combined with each mitochondrial complex separately to quantify the contribution of each complex by explaining V̇O_2peak_ variability. If any of the *r* ^2^ value was greater than 0.75 and thus the variance inflation factor greater than 4.0, we considered multicollinearity might be a problem, but this was not the case in the present analysis. Statistical significance was accepted as *P* < 0.05. Data are presented as mean ± standard deviation. All statistical analyses were performed using GraphPad Prism version 9.0.0.

## Results

### Patient population

Patient characteristics and medications are shown in *Table* *1*, while CPET data are shown in *Table* *2*.

**Table 1:** Clinical variables of patients with heart failure with reduced ejection fraction (n = 22)

Demographics	
Age (years)	69 ± 12
Male sex [*n*(%)]	22 (100)
BMI (kg/m^2^)	28.5 ± 4.0
NYHA Class III/IV [n(%)]	5 (23)
Aetiology of HF	
Ischaemic [*n*(%)]	9 (41)
DCM [*n*(%)]	6 (27)
Past medical history	
Diabetes mellitus [*n*(%)]	6 (27)
AF [*n*(%)]	11 (50)
COPD [*n*(%)]	1 (5)
Hypertension [*n*(%)]	10 (45)
Device therapy	
PPM [*n*(%)]	3 (14)
ICD [*n*(%)]	4 (18)
CRT [*n*(%)]	15 (68)
Medications	
Antiplatelet [*n*(%)]	11 (50)
Beta‐blocker [*n*(%)]	18 (82)
ACE‐I/ARB [*n*(%)]	18 (82)
Loop diuretic [*n*(%)]	11 (50)
MRA [*n*(%)]	11 (50)
Statin [*n*(%)]	16 (73)
Anticoagulant [*n*(%)]	8 (36)
Metformin [*n*(%)]	4 (18)
Insulin [*n*(%)]	2 (9)
Echocardiogram	
LVEF (%)	31.7 ± 13.9
LVEDd (mm)	58.1 ± 7.1
PASP (mmHg)	33.6 ± 15.9
Laboratory investigations	
Hb (g/L)	140.8 ± 15.4
Creatinine (μmol/L)	98.5 ± 24.1
HbA1c (mmol/mol)	45.1 ± 11.1

Normally distributed continuous variables are expressed as mean ± standard deviation; discrete variables are presented as number and percentages in parentheses. ACE‐I, angiotensin converting enzyme inhibitor; ARB, angiotensin II receptor blocker; BMI, body mass index; CHF, chronic heart failure; COPD, chronic obstructive pulmonary disease; CRT, cardiac resynchronisation therapy; DCM, dilated cardiomyopathy; Hb, haemoglobin; HbA1c, glycated haemoglobin; ICD, implantable cardioverter defibrillator; LVED, left ventricular end‐diastolic diameter; LVEF, left ventricular ejection fraction; MRA, mineralocorticoid receptor blocker; NT‐pro‐BNP, N‐terminal pro brain natriuretic peptide; NYHA, New York Heart Association; PASP, pulmonary artery systolic pressure; PPM, permanent pacemaker.

**Table 2:** Exercise variables for patients with heart failure with reduced ejection fraction

CPET variable	Mean ± SD
V̇O_2peak_ (mL/kg/min)	16.7 ± 4.9
V̇O_2LT_ (mL/kg/min)	12.6 ± 4.9
CircP_peak_ (mmHg × mL/kg/min)	2435 ± 863
Peak O_2_ Pulse (mL/beat)	11.9 ± 5.5
RER_peak_	1.1 ± 0.2
VE/VCO_2LT_	32.2 ± 6.1
SBP_peak_ (mmHg)	143.1 ± 22.4
DBP_peak_ (mmHg)	72.0 ± 8.8
HR_peak_ (bpm)	120.1 ± 21.6

CPET, cardiopulmonary exercise testing; peak O_2_ pulse, peak oxygen pulse; CircP_peak_, peak circulatory power; RER_peak_, respiratory exchange ratio at V̇O_2peak_; V̇_E_/V̇CO_2LT_, ventilation and carbon dioxide production ratio at lactate threshold; V̇O_2peak_, peak oxygen uptake; V̇O_2LT_, lactate threshold.

### Correlation between mitochondrial respiration and cardiopulmonary exercise testing variables

**Figure 1:**
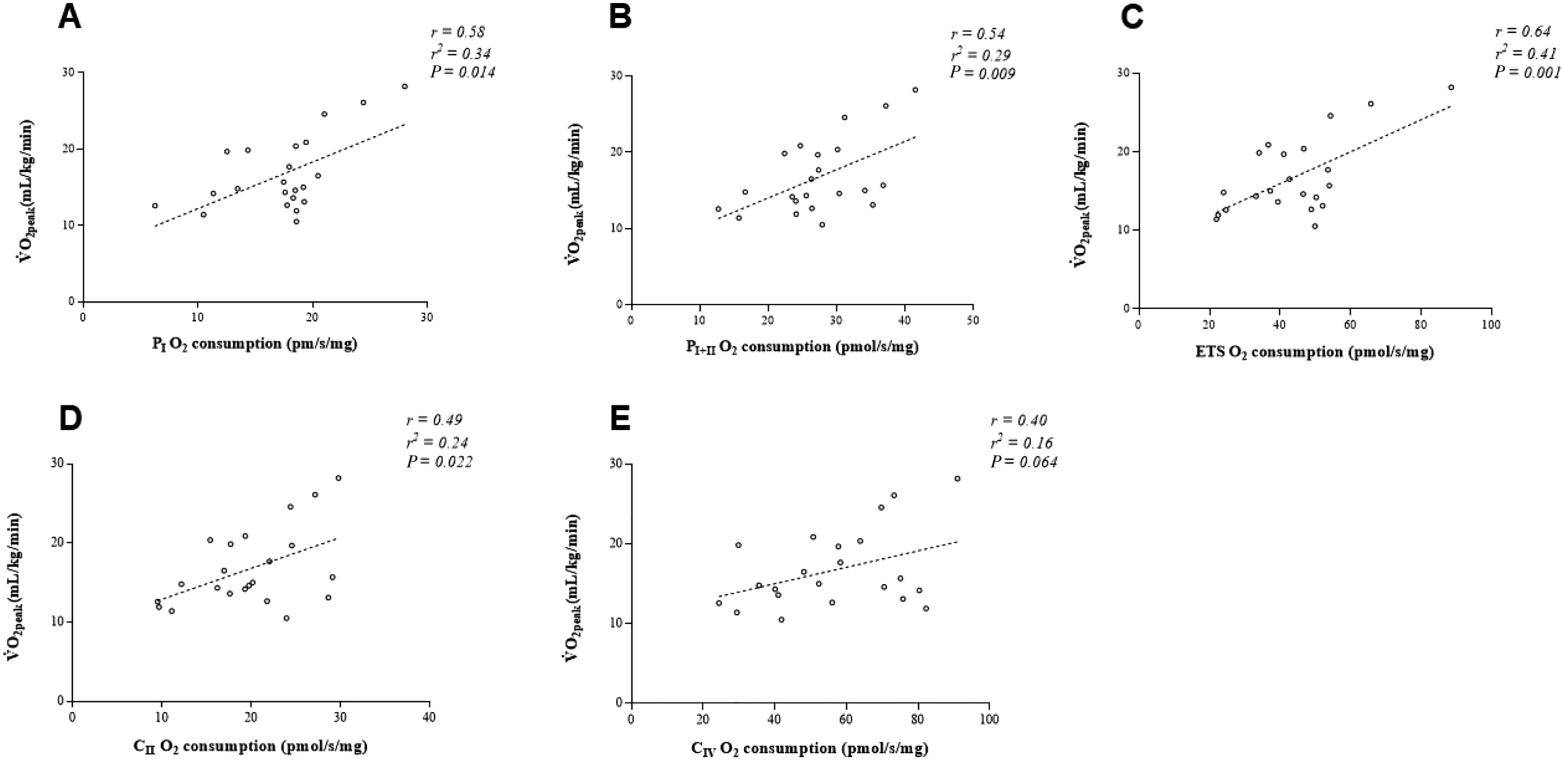
Correlations between mitochondrial respiratory states and whole‐body V̇O_2peak_. P_I_, OXPHOS complex I; P_I + II_, OXPHOS complex; ETS, maximal electron transport system capacity; C_II_, ETS‐supported complex II; C_IV_, complex IV.

We first assessed the correlation between key patient physical and clinical variables with CPET measures. There were no correlations (*P* > 0.05) between age, Hb, and LVEF with V̇O_2peak_ or other CPET variables, although Hb showed a strong trend with V̇O_2peak_ (*r* = 0.38; *P* = 0.08). In contrast, peripheral skeletal muscle mitochondrial function of the *pectoralis major* was well correlated with various whole‐body CPET variables (*Table* *3*). *Figure* *1* displays the correlations between stimulated mitochondrial respiratory states and the most common measure of exercise intolerance: V̇O_2peak_. Mitochondrial respiration of complex I, complex I + II and ETS capacity correlated with V̇O_2peak_ (range: *r* = 0.49–0.64 and *P* < 0.01; *Table* *3* and *Figure* *1A–C*), whereas complexes II and IV did not correlate (*Table* *3*; *Figure* *1D–E*).

**Table 3:** Correlations between *pectoralis major* mitochondrial respiratory states and cardiopulmonary exercise testing variables, including *r*, *r* ^2^ and level of significance

	P_I_	P_I + II_	ETS	C_II_	C_IV_
V̇O_2peak_					
*r*	0.58	0.54	0.64	0.49	0.40
*r* ^2^	0.34	0.29	0.41	0.24	0.16
*P*	0.00	0.01	0.00	0.02	0.06
V̇O_2LT_					
*r*	0.72	0.71	0.70	0.52	0.60
*r* ^2^	0.52	0.49	0.49	0.27	0.36
*P*	0.01	0.01	0.01	0.08	0.04
CircP_peak_					
*r*	0.60	0.56	0.54	0.42	0.49
*r* ^2^	0.36	0.31	0.29	0.18	0.25
*P*	0.01	0.02	0.02	0.08	0.04
Peak O_2_ pulse					
*r*	0.25	0.11	0.31	0.17	0.29
*r* ^2^	0.06	0.01	0.09	0.28	0.08
*P*	0.36	0.69	0.25	0.53	0.28
RER_peak_					
*r*	−0.09	−0.25	−0.12	−0.06	−0.18
*r* ^2^	0.00	0.06	0.01	0.00	0.03
*P*	0.76	0.38	0.68	0.84	0.54
V̇_E_/V̇CO_2LT_					
*r*	−0.15	−0.21	−0.24	−0.48	0.21
*r* ^2^	0.02	0.04	0.06	0.23	0.04
*P*	0.64	0.51	0.45	0.12	0.52

CircP_peak_, peak circulatory power; C_II_, ETS‐supported Complex II; C_IV_, Complex IV; ETS, electron transport system capacity; RER_peak_, respiratory exchange ratio at V̇O_2peak_; V̇_E_/V̇CO_2LT_, ventilation and carbon dioxide production ratio at lactate threshold; V̇O_2peak_, peak oxygen uptake; V̇O_2LT_, lactate threshold; peak O_2_ pulse, peak oxygen pulse; P_I_, OXPHOS Complex I; P_I + II_, OXPHOS complex; *r*, *r* correlation; *r* ^2^, *r* squared; *P*, *P* value.

**Figure 2:**
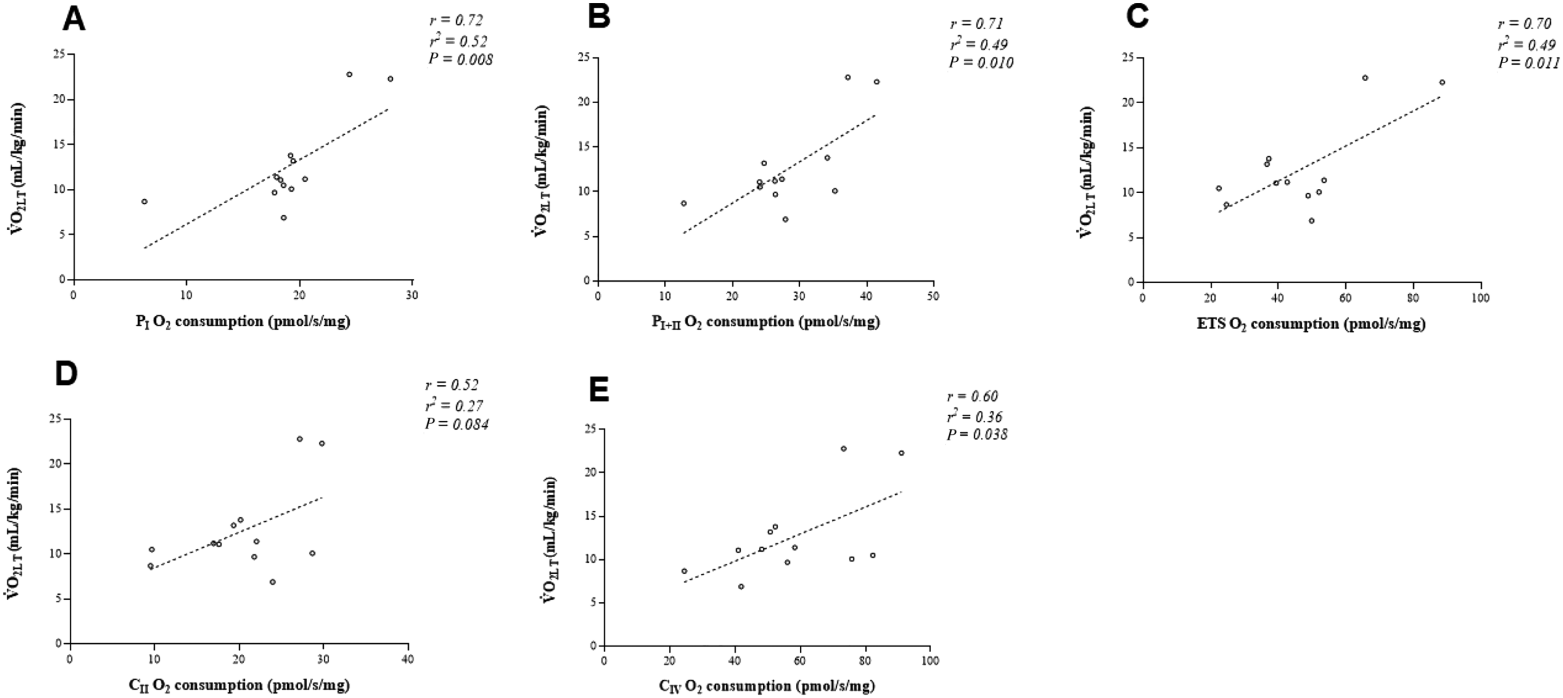
Correlations between mitochondrial respiratory states and lactate threshold (V̇O_2LT_). C_II_, ETS‐supported Complex II; C_IV_, Complex IV; ETS, maximal electron transport system capacity; P_I_, OXPHOS Complex I; P_I + II_, OXPHOS complex.

**Figure 3:**
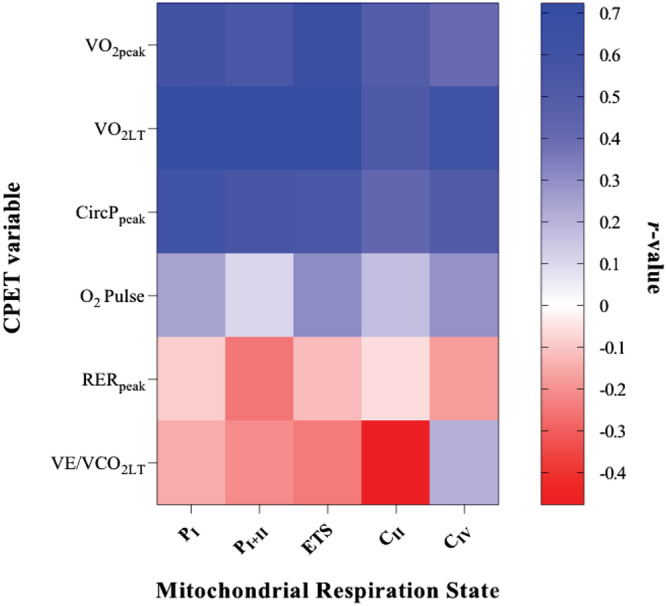
Heat map showing intensity of correlation between mitochondrial respiratory states and whole‐body cardiopulmonary exercise testing (CPET) variables.

We next assessed the correlation between mitochondrial respiration and another key CPET‐derived measure of aerobic performance and prognosis in HFrEF: the V̇O_2LT_ (*Figure* *2*). Mitochondrial respiratory complexes I, I + II, and IV and ETS capacity were all correlated with V̇O_2LT_ (range: *r* = 0.60–0.71 and *P <* 0.05; *Table* *3* and *Figure* *2A–E*), whereas complex II did not (*P* = 0.084; *Table* *3* and *Figure* *2D*). In addition to V̇O_2peak_ and V̇O_2LT_, the V̇E/V̇CO_2LT_ is another strong predictor of prognosis in HFrEF that is thought to be independent of effort. As opposed to V̇O_2peak_ and V̇O_2LT_, we did not observe correlations across mitochondrial respiratory complexes with V̇E/V̇CO_2LT_ (*P* > 0.05; *Table* *3*) or with another variable that is commonly used to indicate maximal effort: RER_peak_ (*P* > 0.05; *Table* *3*). While the peak O_2_ pulse also showed no correlations (*P* > 0.05; *Table* *3*), we found CircP_peak_ was correlated across most mitochondrial respiratory complexes (range: *r* = 0.42–0.60 and *P* < 0.05*; Table* *3*). An overview of the degree of correlation between invasive skeletal muscle mitochondrial measures and non‐invasive whole‐body performance CPET variables is presented in *Figure* *3*, with the strongest relationships found between the mitochondrial respiratory states of ETS capacity and complex I with V̇O_2peak_ and V̇O_2LT_, respectively.

### Multiple regression analysis

**Figure 4:**
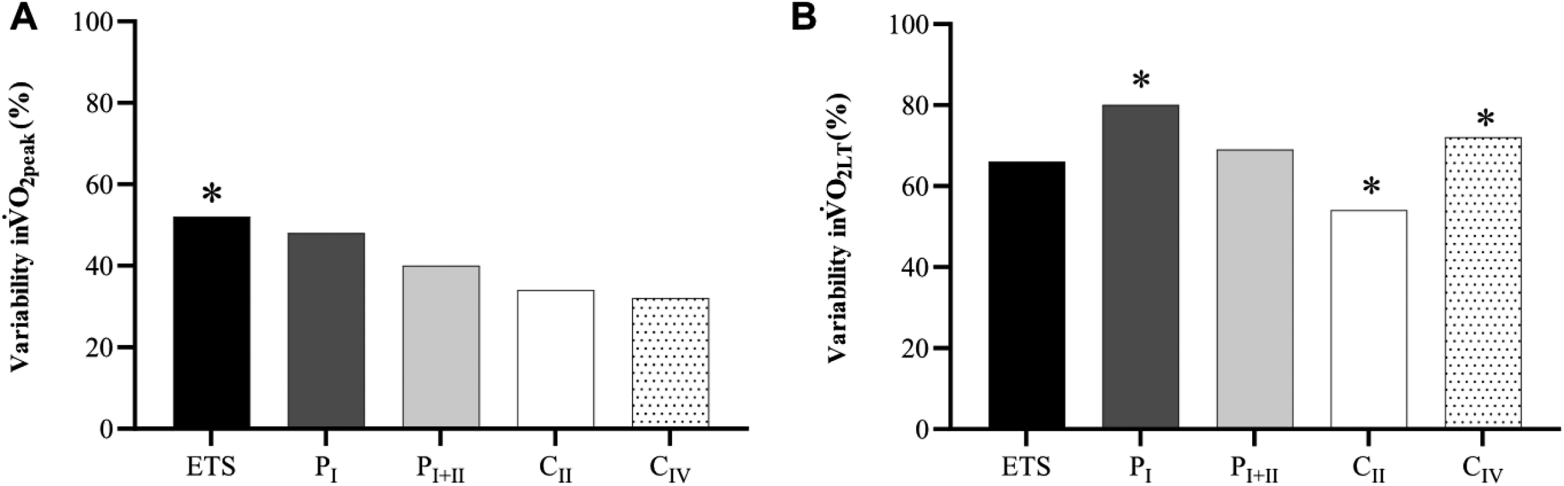
Bar chart demonstrating the percentage V̇O_2peak_ (A) and V̇O_2LT_ (B) explained by individual mitochondrial complexes combined with age, Hb and LVEF. *P < 0.05 for analysis of variance, such that independent variables significantly predict V̇O_2peak_.

A multiple regression was performed with the baseline patient variables including age, Hb, LVEF as one block and alongside each mitochondrial respiratory state in the *pectoralis major* to predict the variability in determining whole‐body V̇O_2peak_ and V̇O_2LT_. Patient characteristics of age, Hb, and LVEF could explain 24% of the variability in V̇O_2peak_; however, the model or one of the contributors did not reach statistical significance. *Figure* *4A* demonstrates the *r* ^2^ value and level of significance for the model combined with each individual mitochondrial complex. Overall, the model that included age, Hb, LVEF, and ETS capacity was able to explain 52% of the variability in V̇O_2peak_ (*r* ^2^ 0.52; *P* = 0.01). From all mitochondrial variables, ETS capacity and complex I were the only independent variables that reached statistical significance while all others resulted in a lower prediction of V̇O_2peak_ (range: 30–50% and *P* > 0.05; *Figure* *4A*). In addition, mitochondrial complex I respiration explained 80% of the variability in V̇O_2LT_ (*r* ^2^ 0.80 and *P* = 0.01; *Figure* *4B*) and complex IV 72% (*r* ^2^ 0.72 and *P* = 0.04; *Figure* *4B*), while the other individual mitochondrial complexes did not reach statistical significance.

## Discussion

We confirm that invasive measurements of mitochondrial respiration from skeletal muscle biopsies of patients with HFrEF independently correlate with standard non‐invasive clinical CPET variables that are commonly used to assess exercise limitation and prognosis. We show that measures of mitochondrial respiration under a range of stimulated conditions (i.e. complexes I, II, and IV and ETS capacity) were correlated with V̇O_2peak_, V̇O_2LT_, and CircP_peak_. In contrast, other important prognostic CPET‐derived indices such as peak O_2_ pulse, V̇_E_/V̇CO_2LT_ and indicators of maximal effort (i.e. RER_peak_) showed no clear relationship. Importantly, when combined with baseline patient characteristics, mitochondrial ETS capacity could explain at least 50% of the variability in V̇O_2peak_ (i.e. one of the strongest measures of exercise limitation and prognosis in HFrEF). Overall, therefore, our findings suggest that therapies targeting specific limitations in mitochondrial respiration related to ETS capacity could benefit whole‐body exercise tolerance and prognosis in patients with HFrEF.

### Is mitochondrial function linked to cardiopulmonary exercise testing variables in heart failure with reduced ejection fraction?

Exercise tolerance depends on the succession of several steps in the oxygen delivery‐utilization cascade from lungs, heart, and vasculature to skeletal muscle mitochondria,^44^ which is most commonly quantified using CPET^42^ where key exercise variables that include V̇O_2peak_, V̇O_2LT_, and V̇_E_/V̇CO_2LT_ strongly predict mortality.^4^ Mitochondrial abnormalities in peripheral skeletal muscle are a major mechanism limiting whole‐body V̇O_2peak_ in patients with HFrEF.^17^, ^18^, ^19^, ^20^, ^21^ Surprisingly few studies have provided a comprehensive assessment of the relationship between mitochondrial function and prognostic CPET variables to delineate mitochondrial‐specific impairments vs. CPET indices beyond V̇O_2peak_. We hypothesized that careful examination of the relationships between invasive mitochondrial and whole‐body CPET variables would provide important insight for understanding markers of exercise limitation and prognosis in HFrEF.

It has previously been shown that static measures of mitochondrial content and morphology are correlated to VO_2peak_ in patients with HFrEF.^25^, ^26^, ^27^, ^28^ Here we uniquely developed this line of enquiry by examining the contribution of dynamic mitochondrial respiration across complexes I, II, and IV and ETS in skeletal muscle to prognostic measures of exercise limitation in patients with HFrEF. Recent detailed assessments of mitochondrial respiration *in situ* have confirmed oxygen flux is lower in patients with HFrEF.^30^, ^31^, ^32^ Impairments to mitochondrial content rather than intrinsic function have been suggested to play the dominant role, with complexe I and IV dysfunction being closely correlated to V̇O_2peak_.^30^, ^31^, ^32^ Earlier studies using more static measurements of mitochondrial function (e.g. *in vitro* enzyme activity or protein content) have also found lower values in patients with HFrEF that correlated to V̇O_2peak_,^25^, ^26^ but findings have been inconsistent in both humans^45^ and animals.^46^, ^47^ However, beyond the V̇O_2peak_, relatively little information has been gleaned from other CPET measures and their relationship to mitochondrial function in HFrEF. This has limited insights into whether mitochondrial abnormalities in HFrEF could also be contributing to other prognostic CPET markers. Here we clearly show that multiple mitochondrial respiration states (e.g. complexes I, II, and IV and ETS) were correlated to the key CPET parameters of V̇O_2LT_ and V̇O_2peak_, but not to the ventilatory prognostic marker of V̇_E_/V̇CO_2LT_. The reason for this is likely explained by both V̇O_2LT_ and V̇O_2peak_ being dictated, in large part, by the degree of oxygen utilization (and thus mitochondrial function), while V̇_E_/V̇CO_2LT_ being more closely linked to pulmonary/acid–base balance coupling.^48^ What is clear is that interventions to improve V̇O_2peak_ in patients with HFrEF are frequently associated with improvements in prognosis: for example, exercise training in patients with HFrEF reverses skeletal mitochondrial abnormalities in parallel to improved V̇O_2peak_ and prognosis.^49^, ^50^, ^51^

One intriguing question, therefore, is whether improving skeletal muscle mitochondrial respiration alone would increase V̇O_2LT_ and V̇O_2peak_ in patients with HFrEF, thereby improving symptoms and prognosis? Rodent models that inherit a genetically high V̇O_2peak_ are known to have higher levels of mitochondrial function in skeletal muscle, and these are linked to reduced development of cardiometabolic disease and improved survival rates.^52^ Thus, these data support the concept that maintaining high ‘mitochondrial fitness’ in skeletal muscle would likely benefit both measures of exercise tolerance and prognosis in patients with HFrEF.

### The contribution of mitochondrial function to exercise limitation in heart failure with reduced ejection fraction

Improving our understanding of the primary factors limiting exercise tolerance in HFrEF will facilitate the identification of more targeted mechanisms and the advent of more effective treatments. Our study and work by others clearly highlight a close relationship between skeletal muscle mitochondrial dysfunction and whole‐body exercise tolerance in HFrEF.^17^, ^18^, ^19^, ^20^, ^21^ However, this study provides further information by using experimental evidence to predict the distinct intrinsic mitochondrial characteristics and their contribution towards the degree of exercise intolerance developed in HFrEF. Here, our findings of the monovariate analysis add new evidence by demonstrating that mitochondrial function under various respiratory states (e.g. P_I_, P_I + II_, and ETS) contributes around 50% and 35% to the variability in VO_2LT_ and VO_2peak_ in HFrEF, respectively. Given that V̇O_2peak_ and V̇O_2LT_ are influenced by other key factors such as cardiac output, blood flow distribution, and oxygen diffusion,^44^ the fact that skeletal muscle mitochondrial function does not entirely explain all the variance in whole‐body V̇O_2peak_ and V̇O_2LT_ is not surprising.

To provide further evidence we also performed a multivariate regression analysis that included key patient characteristics (e.g. age, Hb, and LVEF), which demonstrated that 52% of the variability in V̇O_2peak_ in HFrEF was related to mitochondrial ETS capacity (vs. 41% at the monovariate level). Interestingly only mitochondrial ETS capacity was independently correlated to V̇O_2peak_ in the multivariate analysis and not other respiratory states. These data indicate, therefore, that mitochondrial ETS capacity should be considered one major target for future skeletal muscle mitochondrial therapies in HFrEF, as this would likely achieve the greatest benefits to whole‐body exercise tolerance. The unexplained variance in the model is likely explained by other factors, including inter‐individual pathophysiological differences related to the O_2_ delivery‐utilization cascade related to skeletal muscle, cardiac and pulmonary mechanisms.

Recent data from a larger cohort of patients with HFrEF have also shown that training status and smoking history can further play a role in explaining the V̇O_2peak_ variability.^53^ Indeed, impaired peripheral vascular function is also touted not only to play a major role in causing severe exercise limitations in patients with HFrEF, but as a potentially important upstream mechanism that could mediate severe mitochondrial derangements (i.e. by reducing oxygen delivery via endothelial dysfunction and/or capillary rarefaction).^44^ As such, we acknowledge that mitochondrial respiration cannot entirely explain V̇O_2peak_ variability in HFrEF patients and more research is needed to elucidate other contributing mechanisms using larger sample sizes. Yet, our findings could contribute to better understanding what specific mechanisms allow patients with HFrEF to increase their V̇O_2peak_ following exercise training given the wide variability typically observed, as recently reviewed in detail.^54^

## Conclusion

Invasive measurements of mitochondrial respiratory states from skeletal muscle biopsies of patients with HFrEF were independently correlated to key non‐invasive CPET measures of exercise limitation and prognosis that included V̇O_2peak_, V̇O_2LT_, and CircP_peak_, but not V̇_E_/V̇CO_2LT_, peak O_2_ pulse or RER_peak_. When combined with other patient variables, at least 50% of the variability in V̇O_2peak_ could be explained by the mitochondrial ETS capacity. Overall, these data expand our knowledge on specific mitochondrial targets in the skeletal muscle that may help alleviate symptoms of exercise intolerance in HFrEF.

## Conflict of interest

P. K. declares no conflict of interest. S. S. declares no conflict of interest. J. G. declares no conflict of interest. A. K. declares no conflict of interest. L. D. R. declares no conflict of interest. K. K. W. has received speakers' fees and honoraria from Medtronic, Cardiac Dimensions, Novartis, Abbott, BMS, Pfizer, Bayer, and has received unconditional research grants from Medtronic. C. F. declares no conflict of interest. T. S. B. declares no conflict of interest.

## References

[1] Ross R , Blair SN , Arena R , Church TS , Despres JP , Franklin BA , Haskell WL , Kaminsky LA , Levine BD , Lavie CJ , Myers J , Niebauer J , Sallis R , Sawada SS , Sui X , Wisloff U . Importance of assessing cardiorespiratory fitness in clinical practice: a case for fitness as a clinical vital sign: a scientific statement from the American Heart Association. Circulation 2016; 134: e653–e699. 27881567 10.1161/CIR.0000000000000461

[2] Guazzi M , Arena R , Halle M , Piepoli MF , Myers J , Lavie CJ . 2016 Focused update: clinical recommendations for cardiopulmonary exercise testing data assessment in specific patient populations. Circulation 2016; 133: e694–e711. 27143685 10.1161/CIR.0000000000000406

[3] Guazzi M , Bandera F , Ozemek C , Systrom D , Arena R . Cardiopulmonary exercise testing: what is its value? J Am Coll Cardiol 2017; 70: 1618–1636. 28935040 10.1016/j.jacc.2017.08.012

[4] Tan C , Rossiter HB , Porszasz J , Bowen TS , Witte KK , Stringer WW , Casaburi R , Hansen JE . Reliability and physiological interpretation of pulmonary gas exchange by “circulatory equivalents” in chronic heart failure. J Am Heart Assoc 2018; 7: e008072. 29588313 10.1161/JAHA.117.008072 PMC5907590

[5] Stringer WW . Cardiopulmonary exercise testing: current applications. Expert Rev Respir Med 2010; 4: 179–188. 20406084 10.1586/ers.10.8

[6] Wasserman K , Hansen JE , Sue D , Stringer W , Whipp BJ . Principles of exercise testing and interpretation. Fourth Edition. Philadelphia, PA: Lippincott Williams & Wilkins, 2005.

[7] Yardley M , Havik OE , Grov I , Relbo A , Gullestad L , Nytrøen K . Peak oxygen uptake and self‐reported physical health are strong predictors of long‐term survival after heart transplantation. Clin Transplant 2016; 30: 161–169. 26589579 10.1111/ctr.12672

[8] Brubaker PH , Berry MJ , Brozena SC , Morley DL , Walter JD , Paolone AM , Bove AA . Relationship of lactate and ventilatory thresholds in cardiac transplant patients. Med Sci Sports Exerc 1993; 25: 191–196. 8450720

[9] Keteyian SJ , Patel M , Kraus WE , Brawner CA , McConnell TR , Pina IL , Leifer ES , Fleg JL , Blackburn G , Fonarow GC , Chase PJ , Piner L , Vest M , O'Connor CM , Ehrman JK , Walsh MN , Ewald G , Bensimhon D , Russell SD . Variables measured during cardiopulmonary exercise testing as predictors of mortality in chronic systolic heart failure. J Am Coll Cardiol 2016; 67: 780–789. 26892413 10.1016/j.jacc.2015.11.050 PMC4761107

[10] Gitt AK , Wasserman K , Kilkowski C , Kleemann T , Kilkowski A , Bangert M , Schneider S , Schwarz A , Senges J . Exercise anaerobic threshold and ventilatory efficiency identify heart failure patients for high risk of early death. Circulation 2002; 106: 3079–3084. 12473555 10.1161/01.cir.0000041428.99427.06

[11] Tomono J , Adachi H , Oshima S , Kurabayashi M . Usefulness of anaerobic threshold to peak oxygen uptake ratio to determine the severity and pathophysiological condition of chronic heart failure. J Cardiol 2016; 68: 373–378. 26867779 10.1016/j.jjcc.2016.01.002

[12] Clark AL , Davies LC , Francis DP , Coats AJ . Ventilatory capacity and exercise tolerance in patients with chronic stable heart failure. Eur J Heart Fail 2000; 2: 47–51. 10742703 10.1016/s1388-9842(99)00060-4

[13] Franciosa JA , Park M , Levine TB . Lack of correlation between exercise capacity and indexes of resting left ventricular performance in heart failure. Am J Cardiol 1981; 47: 33–39. 7457405 10.1016/0002-9149(81)90286-1

[14] Witte KK , Nikitin NP , De Silva R , Cleland JG , Clark AL . Exercise capacity and cardiac function assessed by tissue Doppler imaging in chronic heart failure. Heart 2004; 90: 1144–1150. 15367509 10.1136/hrt.2003.025684 PMC1768502

[15] Franciosa JA , Cohn JN . Effect of isosorbide dinitrate on response to submaximal and maximal exercise in patients with congestive heart failure. Am J Cardiol 1979; 43: 1009–1014. 433762 10.1016/0002-9149(79)90368-0

[16] Ponikowski P , Chua TP , Anker SD , Francis DP , Doehner W , Banasiak W , Poole‐Wilson PA , Piepoli MF , Coats AJ . Peripheral chemoreceptor hypersensitivity: an ominous sign in patients with chronic heart failure. Circulation 2001; 104: 544–549. 11479251 10.1161/hc3101.093699

[17] Fülster S , Tacke M , Sandek A , Ebner N , Tschöpe C , Doehner W , Anker SD , Von Haehling S . Muscle wasting in patients with chronic heart failure: results from the studies investigating co‐morbidities aggravating heart failure (SICA‐HF). Eur Heart J 2013; 34: 512–519. 23178647 10.1093/eurheartj/ehs381

[18] Harrington D , Anker SD , Chua TP , Webb‐Peploe KM , Ponikowski PP , Poole‐Wilson PA , Coats AJ . Skeletal muscle function and its relation to exercise tolerance in chronic heart failure. J Am Coll Cardiol 1997; 30: 1758–1764. 9385904 10.1016/s0735-1097(97)00381-1

[19] Lipkin D , Jones DA , Round JM , Poole‐Wilson PA . Abnormalities of skeletal muscle in patients with chronic heart failure. Int J Cardiol 1988; 18: 187–195. 2830194 10.1016/0167-5273(88)90164-7

[20] Massie B , Simonini A , Sahgal P , Wells L , Dudley GA . Relation of systemic and local muscle exercise capacity to skeletal muscle characteristics in men with congestive heart failure. J Am Coll Cardiol 1996; 27: 140–145. 8522687 10.1016/0735-1097(95)00416-5

[21] Schaufelberger M , Eriksson B , Grimby G , Held P , Swedberg K . Skeletal muscle alterations in patients with chronic heart failure. Eur Heart J 1997; 18: 971–980. 9183589 10.1093/oxfordjournals.eurheartj.a015386

[22] Houstis NE , Eisman AS , Pappagianopoulos PP , Wooster L , Bailey CS , Wagner PD , Lewis GD . Exercise intolerance in heart failure with preserved ejection fraction: diagnosing and ranking its causes using personalized O_2_ pathway analysis. Circulation 2018; 137: 148–161. 28993402 10.1161/CIRCULATIONAHA.117.029058 PMC5760316

[23] Kumar AA , Kelly DP , Chirinos JA . Mitochondrial dysfunction in heart failure with preserved ejection fraction. Circulation 2019; 139: 1435–1450. 30856000 10.1161/CIRCULATIONAHA.118.036259 PMC6414077

[24] Haykowsky MJ , Tomczak CR , Scott JM , Paterson DI , Kitzman DW . Determinants of exercise intolerance in patients with heart failure and reduced or preserved ejection fraction. J Appl Physiol 2015; 119: 739–744. 25911681 10.1152/japplphysiol.00049.2015 PMC4687865

[25] Drexler H , Riede U , Munzel T , Konig H , Funke E . Just H. Alterations of skeletal muscle in chronic heart failure. Circulation 1992; 85: 1751–1759. 1315220 10.1161/01.cir.85.5.1751

[26] Garnier A , Fortin D , Zoll J , N'Guessan B , Mettauer B , Lampert E , Veksler V , Ventura‐Clapier R . Coordinated changes in mitochondrial function and biogenesis in healthy and diseased human skeletal muscle. FASEB J 2005; 19: 43–52. 15629894 10.1096/fj.04-2173com

[27] Hambrecht R , Fiehn E , Yu J , Niebauer J , Weigl C , Hilbrich L , Adams V , Riede U , Schuler G . Effects of endurance training on mitochondrial ultrastructure and fiber type distribution in skeletal muscle of patients with stable chronic heart failure. J Am Coll Cardiol 1997; 29: 1067–1073. 9120161 10.1016/s0735-1097(97)00015-6

[28] Larsen AI , Lindal S , Myreng K , Ogne C , Kvaloy JT , Munk PS , Aukrust P , Yndestad A , Dickstein K , Nilsen DW . Cardiac resynchronization therapy improves minute ventilation/carbon dioxide production slope and skeletal muscle capillary density without reversal of skeletal muscle pathology or inflammation. Europace 2013; 15: 857–864. 23322010 10.1093/europace/eus428

[29] Pesta D , Gnaiger E . High‐resolution respirometry: OXPHOS protocols for human cells and permeabilized fibers from small biopsies of human muscle. Methods Mol Biol 2012; 810: 25–58. 22057559 10.1007/978-1-61779-382-0_3

[30] Caspi T , Straw S , Cheng C , Garnham JO , Scragg JL , Smith J , Koshy AO , Levelt E , Sukumar P , Gierula J , Beech DJ , Kearney MT , Cubbon RM , Wheatcroft SB , Witte KK , Roberts LD , Bowen TS . Unique transcriptome signature distinguishes patients with heart failure with myopathy. J Am Heart Assoc 2020; 9: e017091. 32892688 10.1161/JAHA.120.017091 PMC7727001

[31] Garnham JO , Roberts LD , Caspi T , Al‐Owais MM , Bullock M , Swoboda PP , Koshy A , Gierula J , Paton MF , Cubbon RM , Kearney MT , Bowen TS , Witte KK . Divergent skeletal muscle mitochondrial phenotype between male and female patients with chronic heart failure. J Cachexia Sarcopenia Muscle 2020; 11: 79–88. 31430834 10.1002/jcsm.12488 PMC7015245

[32] Garnham JO , Roberts LD , Espino‐Gonzalez E , Whitehead A , Swoboda PP , Koshy A , Gierula J , Paton MF , Cubbon RM , Kearney MT , Egginton S , Bowen TS , Witte KK . Chronic heart failure with diabetes mellitus is characterized by a severe skeletal muscle pathology. J Cachexia Sarcopenia Muscle 2020; 11: 394–404. 31863644 10.1002/jcsm.12515 PMC7113493

[33] Ponikowski P , Voors AA , Anker SD , Bueno H , Cleland JG , Coats AJ , Falk V , Gonzalez‐Juanatey JR , Harjola VP , Jankowska EA , Jessup M , Linde C , Nihoyannopoulos P , Parissis JT , Pieske B , Riley JP , Rosano GM , Ruilope LM , Ruschitzka F , Rutten FH , van der Meer P . 2016 ESC Guidelines for the diagnosis and treatment of acute and chronic heart failure: The Task Force for the diagnosis and treatment of acute and chronic heart failure of the European Society of Cardiology (ESC). Developed with the special contribution of the Heart Failure Association (HFA) of the ESC. Eur J Heart Fail 2016; 18: 891–975. 27207191 10.1002/ejhf.592

[34] Beaver WL , Wasserman K , Whipp BJ . A new method for detecting anaerobic threshold by gas exchange. J Appl Physiol (1985) 1986; 60: 2020–2027. 3087938 10.1152/jappl.1986.60.6.2020

[35] Tang Y , Yao L , Liu Z , Xie W , Ma X , Luo Q , Zhao Z , Huang Z , Gao L , Jin Q , Yu X , Xiong C , Ni X , Yan Y , Qi W . Peak circulatory power is a strong prognostic factor in patients with idiopathic pulmonary arterial hypertension. Respir Med 2018; 135: 29–34. 29414450 10.1016/j.rmed.2018.01.003

[36] Cohen‐Solal A , Tabet JY , Logeart D , Bourgoin P , Tokmakova M , Dahan M . A non‐invasively determined surrogate of cardiac power (‘circulatory power’) at peak exercise is a powerful prognostic factor in chronic heart failure. Eur Heart J 2002; 23: 806–814. 12009721 10.1053/euhj.2001.2966

[37] Nicholls DP , O'Dochartaigh C , Riley MS . Circulatory power—a new perspective on an old friend. Eur Heart J 2002; 23: 1242–1245. 12175661 10.1053/euhj.2002.3229

[38] Tabet JY , Metra M , Thabut G , Logeart D , Cohen‐Solal A . Prognostic value of cardiopulmonary exercise variables in chronic heart failure patients with or without beta‐blocker therapy. Am J Cardiol 2006; 98: 500–503. 16893705 10.1016/j.amjcard.2006.03.027

[39] Kumar A , Ansari BA , Kim J , Suri A , Gaddam S , Yenigalla S , Vanjarapu JM , Selvaraj S , Tamvada D , Lee J , Akers SR , Chirinos JA . Axial muscle size as a strong predictor of death in subjects with and without heart failure. J Am Heart Assoc 2019; 8: e010554. 30755074 10.1161/JAHA.118.010554 PMC6405649

[40] Kuznetsov AV , Gnaiger E . Oxygraph assay of cytochrome c oxidase activity: chemical background correction. Mitochondr Phys Netw 2010; 6: 1–4.

[41] di Prampero PE , Ferretti G . Factors limiting maximal oxygen consumption in humans. Respir Physiol 1990; 80: 113–128. 2218094 10.1016/0034-5687(90)90075-a

[42] Bassett DR Jr , Howley ET . Limiting factors for maximum oxygen uptake and determinants of endurance performance. Med Sci Sports Exerc 2000; 32: 70–84. 10647532 10.1097/00005768-200001000-00012

[43] Levine BD . What do we know, and what do we still need to know? J Physiol 2008; 586: 25–34. 18006574 10.1113/jphysiol.2007.147629 PMC2375567

[44] Poole DC , Hirai DM , Copp SW , Musch TI . Muscle oxygen transport and utilization in heart failure: implications for exercise (in)tolerance. Am J Physiol Heart Circ Physiol 2012; 302: H1050–H1063. 22101528 10.1152/ajpheart.00943.2011 PMC3311454

[45] Williams AD , Selig S , Hare DL , Hayes A , Krum H , Patterson J , Geerling RH , Toia D , Carey MF . Reduced exercise tolerance in CHF may be related to factors other than impaired skeletal muscle oxidative capacity. J Card Fail 2004; 10: 141–148. 15101026 10.1016/j.cardfail.2003.09.004

[46] Garnier A , Fortin D , Delomenie C , Momken I , Veksler V , Ventura‐Clapier R . Depressed mitochondrial transcription factors and oxidative capacity in rat failing cardiac and skeletal muscles. J Physiol 2003; 551: 491–501. 12824444 10.1113/jphysiol.2003.045104 PMC2343221

[47] De Sousa E , Veksler V , Bigard X , Mateo P , Ventura‐Clapier R . Heart failure affects mitochondrial but not myofibrillar intrinsic properties of skeletal muscle. Circulation 2000; 102: 1847–1853. 11023942 10.1161/01.cir.102.15.1847

[48] Whipp BJ . Physiological mechanisms dissociating pulmonary CO2 and O2 exchange dynamics during exercise in humans. Exp Physiol 2007; 92: 347–355. 17185348 10.1113/expphysiol.2006.034363

[49] Cattadori G , Segurini C , Picozzi A , Padeletti L , Anzà C . Exercise and heart failure: an update. ESC Heart Fail 2018; 5: 222–232. 10.1002/ehf2.12225 PMC5880674 29235244

[50] Flynn KE , Piña IL , Whellan DJ , Lin L , Blumenthal JA , Ellis SJ , Fine LJ , Howlett JG , Keteyian SJ , Kitzman DW , Kraus WE , Miller NH , Schulman KA , Spertus JA , O'Connor CM , Weinfurt KP , Investigators H‐A . Effects of exercise training on health status in patients with chronic heart failure: HF‐ACTION randomized controlled trial. JAMA 2009; 301: 1451–1459. 19351942 10.1001/jama.2009.457 PMC2690699

[51] Rees K , Taylor RS , Singh S , Coats AJS , Ebrahim S . Exercise based rehabilitation for heart failure. Cochrane Database Syst Rev 2004: CD003331–CD003331. 15266480 10.1002/14651858.CD003331.pub2 PMC4164468

[52] Wisløff U , Najjar SM , Ellingsen O , Haram PM , Swoap S , Al‐Share Q , Fernström M , Rezaei K , Lee SJ , Koch LG , Britton SL . Cardiovascular risk factors emerge after artificial selection for low aerobic capacity. Science 2005; 307: 418–420. 15662013 10.1126/science.1108177

[53] Karlsen T , Videm V , Halle M , Ellingsen O , Stoylen A , Dalen H , Delagardelle C , Larsen AI , Hole T , Mezzani A , Craenenbroeck EMV , Beckers P , Pressler A , Christle JW , Winzer EB , Mangner N , Woitek FJ , Hollriegel R , Snoer M , Feiereisen P , Valborgland T , Linke A , Prescott E . Baseline and exercise predictors of V O_2_ peak in systolic heart failure patients: results from SMARTEX‐HF. Med Sci Sports Exerc 2020; 52: 810–819. 31688648 10.1249/MSS.0000000000002193

[54] Gevaert AB , Adams V , Bahls M , Bowen TS , Cornelissen V , Dörr M , Hansen D , Kemps HM , Leeson P , Van Craenenbroeck EM , Kränkel N . Towards a personalised approach in exercise‐based cardiovascular rehabilitation: How can translational research help? A ‘call to action’ from the Section on Secondary Prevention and Cardiac Rehabilitation of the European Association of Preventive Cardiology. Eur J Prev Cardiol 2020; 27: 1369–1385. 31581819 10.1177/2047487319877716

